# Endothelin-1 precursor peptides correlate with severity of disease and outcome in patients with community acquired pneumonia

**DOI:** 10.1186/1471-2334-8-22

**Published:** 2008-02-28

**Authors:** Philipp Schuetz, Daiana Stolz, Beat Mueller, Nils G Morgenthaler, Joachim Struck, Christian Mueller, Roland Bingisser, Michael Tamm, Mirjam Christ-Crain

**Affiliations:** 1 Department of Internal Medicine, University Hospital Basel, Switzerland; 2 Clinic of Pneumology and Pulmonary Cell Research, University Hospital Basel, Switzerland; 3 Research Department, B.R.A.H.M.S AG, Biotechnology Center Hennigsdorf/Berlin, Germany

## Abstract

**Background:**

Circulating levels of endothelin-1 are increased in sepsis and correlate with severity of disease. A rapid and easy immunoassay has been developed to measure the more stable ET-1 precursor peptides proET-1. The objective of this study was to assess the diagnostic and prognostic value of proET-1 in a prospective cohort of mainly septic patients with community-acquired pneumonia.

**Methods:**

We evaluated 281 consecutive patients with community acquired pneumonia. Serum proET-1 plasma levels were measured using a new sandwich immunoassay.

**Results:**

ProET-1 levels exhibited a gradual increase depending on the clinical severity of pneumonia as assessed by the pneumonia severity index (PSI) and the CURB65 scores (p < 0.001 and p < 0.01). The diagnostic accuracy to predict bacteraemia of procalcitonin (AUC 0.84 [95% 0.74–0.93]) was superior than C-reactive protein (AUC 0.67 [95%CI 0.56–0.78]) and leukocyte count (AUC 0.66 [95%CI 0.55–0.78]) and in the range of proET-1(AUC of 0.77 [95%CI 0.67–0.86]). ProET-1 levels on admission were increased in patients with adverse medical outcomes including death and need for ICU admission. ROC curve analysis to predict the risk for mortality showed a prognostic accuracy of proET-1 (AUC 0.64 [95%CI 0.53–0.74]), which was higher than C-reactive protein (AUC 0.51 [95%CI 0.41–0.61]) and leukocyte count (AUC 0.55 [95%CI 0.44–0.65]) and within the range of the clinical severity scores (PSI AUC 0.69 [95%CI 0.61–0.76] and CURB65 0.67 [95%CI 0.57–0.77]) and procalcitonin (AUC 0.59 [95% 0.51–0.67]). ProET-1 determination improved significantly the prognostic accuracy of the CURB65 score (AUC of the combined model 0.69 [95%CI 0.59–0.79]). In a multivariate logistic regression model, only proET1 and the clinical severity scores were independent predictors for death and for the need for ICU admission.

**Conclusion:**

In community-acquired pneumonia, ET-1 precursor peptides correlate with disease severity and are independent predictors for mortality and ICU admission. If confirmed in future studies, proET-1 levels may become another helpful tool for risk stratification and management of patients with community-acquired pneumonia.

**Trial registration:**

ISRCTN04176397

## Background

Endothelin-1 (ET-1) is a potent vasoconstrictor agent, synthesized mainly by endothelial cells [1,2]. In the experimental setting, endotoxaemia induces the expression of endothelin precursors (prepro-Endothelin) mRNA in the heart and the lung [2,3]. In humans, elevated plasma levels of mature ET-1 are found during systemic infections and increased plasma ET-1 levels correlate with mortality risk [3-5]. In addition, animal studies demonstrated beneficial effects of ET-1 antagonism by using a selective ET receptor antagonist during septic shock [6-9]. Regrettably, the analytical reliability of ET-1 measurements is cumbersome because it is instable at room temperature and is rapidly cleared from the circulation limiting its use in clinical routine. Recently, a new sandwich immunoassay has been introduced that measures the more stable precursor fragments proET-1 [10,11]. Unlike the mature peptide, these precursors can be detected for hours in the circulation. Because of the stoichiometric generation, this "prohormone" correlates with the release of the active peptide [10], a condition similar to that of insulin and C-peptide. Thus, these precursor peptides can be used to indirectly measure the release of mature ET-1 in physiological and pathological conditions.

At present there are no clinical data available regarding the release of proET-1 during severe systemic infections other than sepsis[12]. As community-acquired pneumonia (CAP) is the most important precursor of sepsis, we hypothesize that circulating proET-1 levels are increased during the acute illness and might predict adverse outcome in a well-defined cohort of 281 patients with CAP requiring hospitalization.

## Methods

### Setting and Study population

The present study evaluated data and available plasma samples from 281 patients admitted to the emergency department with CAP from November 2003 through February 2005 [13]. The primary endpoint of the study was antibiotic stewardship guided by procalcitonin as compared to standard recommended guidelines [13]. A predefined secondary endpoint was the assessment of prognostic factors and biomarkers in CAP. A detailed description of the study has been published elsewhere [13]. Briefly, patients admitted to the University Hospital Basel, Switzerland, a 950-bed tertiary care hospital with suspected CAP and age > 18 years were consecutively included in this study. Excluded were patients with cystic fibrosis, active pulmonary tuberculosis, hospital-acquired pneumonia and patients with severe immuno-suppression. Patients were examined on admission to the emergency department by a resident supervised by a board-certified specialist in Internal Medicine. Baseline assessment included clinical data and vital signs, assessment of patients' functional status using a visual analogue scale, comorbid conditions, and routine blood tests. In all patients, the Pneumonia Severity Index (PSI) and the CURB65 score were calculated [14]. Forty-nine percent of the patients [N = 138] were randomized to receive antibiotic treatment according to procalcitonin guidance and 51% patients [N = 143] were allocated to the control group. Treatment allocation did not have a significant impact on all-cause mortality (OR 0.85 [95%CI: 0.42–1.74], p = 0.67) or admission to the ICU (OR 0.85 [95%CI: 0.48–1.49], p = 0.571) and was thus not considered any further in this analysis.

CAP was defined by the presence of recently acquired respiratory signs, core body temperature >38.0°C, auscultatory findings of abnormal breath sounds or rales, leukocyte count >10 or <4 × 10^9 ^cells L^-1 ^and an infiltrate on chest radiograph [15]. Chest radiographs were screened by the physician in charge and reviewed by a senior radiologist, unaware of clinical and laboratory findings.

The study was approved by the institutional review board (Ethikkommission beider Basel, EKBB) and written informed consent was obtained from all included patients. All data were held and analyzed by the authors.

### Outcome

For outcome assessment, a follow up examination was planned 6 weeks after study inclusion. Patients were followed-up for a median duration of 42 days [IQR 35–53]. Two patients did not complete the follow up and as the resolution of illness was uneventful during the hospitalisation, the day of discharge was counted as the time point of follow up. Patients who survived until follow-up were counted as survivors, whereas patients who died within the follow-up period were counted as non-survivors. Adverse medical outcome for this analysis was defined as death and need for Intensive Care Unit (ICU) admission from any cause.

### Microbial investigations

The laboratory workup for the patients with CAP has been previously described [13]. Briefly, it included sputum samples for Gram stain and culture, two blood samples for culture and urine sample for detection of *Legionella pneumophila*.

### Measurements of proET-1 and other laboratory parameters

Pro-ET1 was batch-measured in the plasma with a new sandwich immunoassay as described elsewhere (CT-proET1, BRAHMS AG, Hennigsdorf, Berlin, Germany) [10,11]. The assay (normal reference range 44.3 ± 10.6 pmol/l) has an analytical detection limit of 0.4 pmol/l. C-reactive protein was measured in EDTA plasma on a Hitachi Instrument 917 (Roche Diagnostics, Rotkreuz, Switzerland). Procalcitonin was measured using 20 to 50 μL of plasma or serum by a time-resolved amplified cryptate emission (TRACE) technology assay (PCT Kryptor^®^, B.R.A.H.M.S. AG, Hennigsdorf, Germany). The assay has a functional assay sensitivity of 0.06 μg/L, 3 to 10-fold above normal mean values.

### Statistical analysis

Discrete variables are expressed as counts (percentage) and continuous variables as medians and interquartile Ranges (IQR) unless stated otherwise. Frequency comparison was done by chi-square test. Two-group comparison of normally distributed data was performed by Students t-test. For multigroup comparisons, one-way analysis of variance with least square difference for posthoc comparison was applied. For data not normally distributed, the Mann-Whitney-U test was used if only two groups were compared and the Kruskal-Wallis one-way analysis of variance was used if more than two groups were being compared. Receiver-operating-characteristics were calculated using STATA 9.2 (Stata Corp, College Station, Tex). Thereby, outcomes were either survival until follow-up or adverse medical outcome including death and need for ICU admission until follow-up, respectively. To estimate the potential clinical relevance of proET-1 measurements, we used likelihood-ratio tests to determine whether logistic regression models that included measurements of proET-1 and PSI/CURB65 provided a significant better fit than did logistic regression models limited to the PSI or CURB65 alone. Correlation analyses were performed by using Spearman rank correlation. Levels that were non-detectable were assigned a value equal to the lower limit of detection for the assay. All testing was two-tailed and P values less than 0.05 were considered to indicate statistical significance.

## Results

### Baseline parameters

The median age of the 281 patients was 74 years and 20 percent of patients were pretreated with antibiotics. Temperature ≥ 38°C was present in 64 percent of patients. Cough, increased sputum production and dyspnea, the typical self-reported cardinal symptoms of CAP, were present in 89, 74 and 75 percent of all patients, respectively. Overall, 80 percent of patients had relevant comorbidities including chronic obstructive pulmonary disease in 25 percent and coronary or hypertensive cardiopathy in 53 percent. The median PSI score at presentation to the Emergency Department was 100 [IQR 77–124] points. 39 percent of all patients were in the low risk PSI classes 1, 2 and 3. The median CURB65 score was 2 [IQR 2–3] and 47 percent of patients were in the low risk classes 0 and 1. 97 percent of patients were hospitalized for more than one night with a median length of hospital stay of 11 days [IQR 7–17]. Blood culture collection was performed in 89 percent of patients and in 12 percent growth of microorganisms was observed (*S. pneumoniae *(60%), *S. aureus *(10%), *E. coli *(6%), *Klebsiella pneumoniae (*6%)). Detailed baseline characteristics of the study population are summarized in Table 1.

**Table 1:** Baseline Characteristics of the 281 Patients with CAP

***Demographic characteristics***	
	
**Age **– years – [median-IQR]	74 [61–82]
**Male sex **– no. [%]	175 [62]
**Coexisting illnesses – no. [%]**	
-Heart disease	149 [53]
-Renal dysfunction	76 [27]
-Chronic obstructive pulmonary disease	71 [25]

***Clinical and laboratory findings***	
	
**Clinical examination**	
-Confusion – no. [%]	25 [9]
-Systolic blood pressure mmHg- [median-IQR]	130 [112–142]
-Heart rate [bpm] – [median-IQR]	96 [82–110]
-Temperature [C°] – [median-IQR]	38.4 [37.7–39.2]

**Laboratory findings **– [median-IQR]	
-C-reactive protein [mg/L)	132 [67–212]
-Procalcitonin [μg/L)	0.52 [0.20–2.45]
-Leukocyte count [× 10^9^)	12.8 [9.1–16.8]
-ProET-1 [nmol/L)	94.6 [65.5–139.0]

***Risk assessment***	
	
**PSI points **– [median-IQR]	100 [77–124]
**PSI class **– [median-IQR]	4 (3–4)
**CURB65 **– [median-IQR]	2 (1–2)

IQR denotes Interquartile Range, PSI Pneumonia Severity Index

### ProET1 levels at presentation and recovery and correlation with disease severity

**Figure 1:**
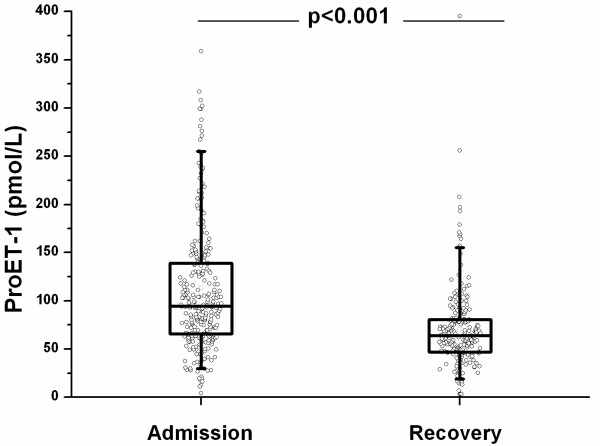
ProET-1 levels on admission and during/after recovery after 42 days [IQR 35–53]. Data are shown as box plots.

**Figure 2:**
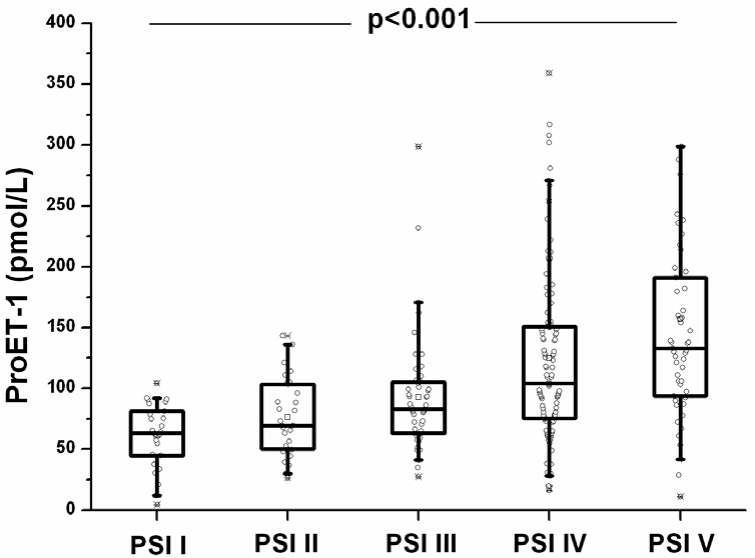
ProET-1 levels increase according to disease severity as represented by the PSI (Pneumonia Severity Index).

**Figure 3:**
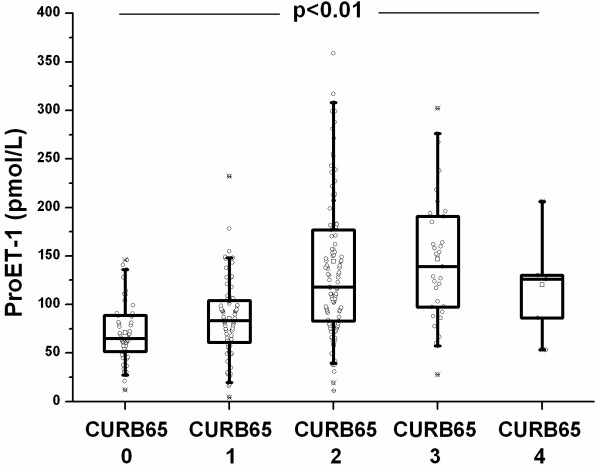
ProET-1 levels increase according to disease severity as represented by the CURB65 score (Confusion – Urea – Respiration rate – Blood pressure – Age 65).

ProET1 (pmol/L) levels were significantly higher at hospital admission as compared to recovery (108.1 [IQR 99.3–117.0] vs 70.1 [IQR 64.6–71.5], p < 0.0001 (Figure 1). On admission, pro-ET1 levels significantly increased with increasing severity of CAP as determined by the PSI scores (Figure 2) and the CURB65 (Figure 3) score (p < 0.001 and p < 0.01). Median proET1 levels showed an about 2-fold increase from patients with PSI class 1 to PSI class 5, and a 0.4-fold increase from CURB65 class 0 to class 4, respectively. This gradual increase was also present for procalcitonin levels (p < 0.01, p < 0.01) and total leukocyte count (p = 0.03, p = 0.004), but not for C-reactive protein (p = 0.12, p = 0.61) and body temperature (p = 0.59, p = 0.42).

ProET-1 levels showed significant correlations with renal function (serum creatinine (r^2 ^= 0.29, p < 0.0001) and urea levels (r^2 ^= 0.32, p < 0.0001)).

### Pro-ET1 levels as diagnostic markers of bacterial infection

ProET-1 (pmol/L) levels were significantly higher in patients with growth of bacteria in their blood culture (n = 31) as compared to patients without growth of bacteria (160.0 [IQR 95.7–218.0] vs 92.4 [IQR 63.7–128.5], p < 0.0001). ProET-1 levels significantly correlated with other biomarkers of infection, i.e. procalcitonin (r^2 ^= 0.31, p < 0.0001), C-reactive protein (r^2 ^= 0.11, p < 0.0001) and total leukocyte count (r^2 ^= 0.07, p < 0.001).

The diagnostic accuracy of procalcitonin to predict bacteraemia (AUC 0.84 [95% 0.74–0.93]) was in the range of ProET1 (AUC of 0.77 [95%CI 0.67–0.86], p = 0.21) and superior than C-reactive protein (AUC 0.67 [95%CI 0.56–0.78], p = 0.004) and leukocyte count (AUC0.66 [95%CI 0.55–0.78], p = 0.03). At an optimal cut-off of 154 pmol/L, proET-1 had a sensitivity and specificity of 62% and 87% to predict bacteraemia and a positive and negative predictive value of 37% and 95%. Likewise, procalcitonin had a sensitivity and specificity of 86% and 74% and a positive and negative predictive value of 27% and 98% at an optimal cut-off of 1.34 μg/L.

### Pro-ET1 levels as prognostic markers for outcome

**Figure 4:**
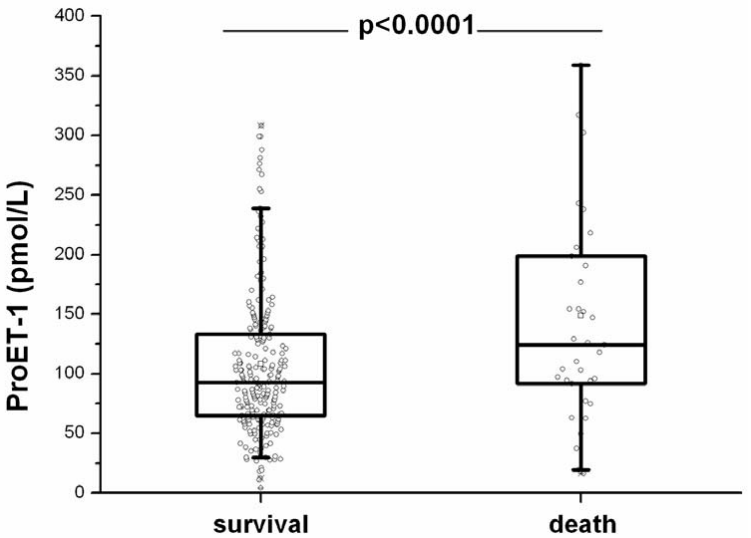
ProET-1 levels in survivors and nonsurvivors.

**Figure 5:**
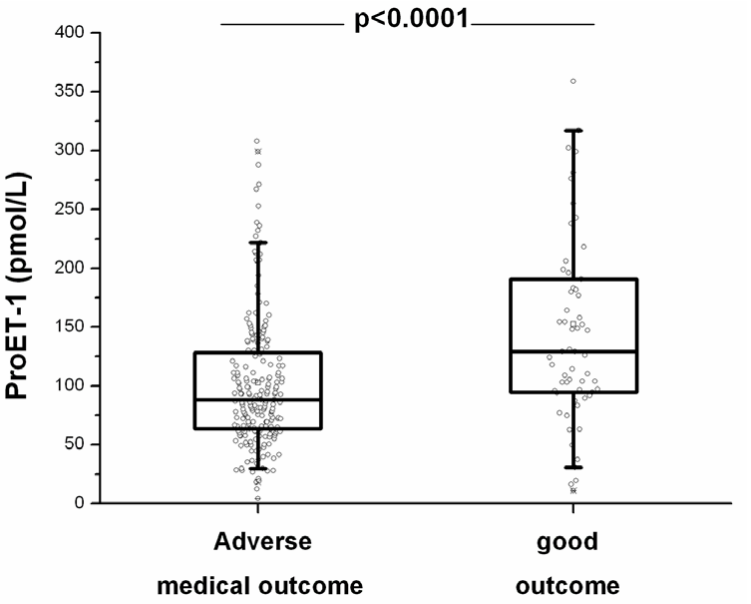
ProET-1 levels in patients with and without an adverse medical outcome including death and/or need for ICU admission.

At follow-up, an adverse medical outcome was noted in 61 patients (22%), including 35 deaths (13%) and 36 admissions to the ICU (13%). The reason for ICU relocations were need for invasive (n = 7) and non-invasive (n = 17) ventilation and hemodynamic stabilization because of sepsis related hypotension (n = 12). Ten patients who were admitted to the ICU subsequently died. In patients who died during follow-up, proET-1 (pmol/L) levels on admission were significantly higher as compared to levels in survivors (124.0 [IQR 91.7–199.0] vs. 92.9 [IQR 65.0–133.0], p = 0.008) (Figure 4). The respective values for other markers of infection were not significant (for procalcitonin: 0.61 [IQR 0.37–3.13] vs. 0.48 [IQR 0.18–2.45] μg/L (p = 0.09), for C-reactive protein 152 [IQR 84–211] vs. 132 [IQR 66–213] mg/L (p = 0.83) and for total leukocyte count (13.5 [IQR 10.3–16.9] vs. 12.7 [IQR 9.0–16.7] × 10^9^/L (p = 0.35)). Moreover, proET1 levels but not procalcitonin, C-reactive protein and leukocyte count were elevated in patients with an adverse medical outcome consisting of either death or ICU admission (129.0 [IQR 94.6–191.0] vs. 88.2 [IQR 63.4–128.0] pmol/L, p < 0.0001) (Figure 5).

To assess the prognostic ability of proET-1 to predict (a) death and (b) adverse medical outcome including death and ICU admission, a receiver operating curve (ROC) was calculated. The discriminatory ability to predict death and adverse outcome of proET1 (AUC 0.64 [95%CI 0.53–0.74] and AUC 0.69 [95% 0.61–0.77]) was significantly better as compared to C-reactive protein (AUC 0.51 [95% 0.41–0.61] and AUC 0.58 [95% 0.51–0.66]) and leukocyte count (AUC 0.55 [95% 0.44–0.65] and AUC 0.57 [95% 0.49–0.65]) and tended to be better as compared to procalcitonin (AUC 0.59 [95% 0.51–0.67] and AUC 0.65 [95% 0.57–0.72]). As demonstrated in Table 2, the prognostic accuracy of proET-1 was in the range of both clinical assessment scores (PSI and CURB65). The optimal prognostic accuracy for pro-ET1 was at a cut-off of 94 pmol/L. With this cut-off, the sensitivity to correctly predict mortality until follow-up was 71 percent, the specificity 53 percent, the positive likelihood ratio (LHR+) 1.5 and the negative likelihood ratio (LHR-) 0.54. The respective values to predict adverse medical outcome were seventy-seven, fifty-six percent and 1.8 and 0.4, respectively.

**Table 2:** Results of the receiver operating curve (ROC) characteristic plot analysis

**Prediction of death **(*n *= *35*) **in patients with CAP **(*n *= *281*)			
**Parameter**	**AUC**	**95% CI**	***P ***

ProET-1	0.64	0.53–0.74	
PSI score	0.69	0.61–0.76	*0.32*
PSI/proET-1 *combined*	0.71	0.62–0.76	*0.04*
CURB65 score	0.67	0.57–0.77	*0.51*
CURB65/proET-1 *combined*	0.69	0.59–0.79	*0.16*

**Prediction of adverse outcome **(*n *= *61*) **in patients with CAP **(*n *= *281*)			

**Parameter**	**AUC**	**95% CI**	***P ***

ProET-1	0.69	0.61–0.77	
PSI classes	0.71	0.65–0.78	*0.57*
PSI/proET-1 *combined*	0.75	0.68–0.81	*0.06*
CURB65 score	0.66	0.58–0.73	*0.45*
CURB65/proET-1 *combined*	0.69	0.62–0.77	*0.75*

CAP Community-acquired Pneumonia, AUC, Area Under the Curve; CI, Confidence Interval, PSI Pneumonia Severity Index, CURB65 Confusion – Urea – Respiration rate – Blood pressure – Age 65

To estimate the additive value of proET-1 on the two clinical scores to predict death and death and/or ICU admission, we calculated a multivariate logistic regression model combing the PSI and proET1 and the CURB65 and proET-1, respectively (Table 2). ProET-1 improved the CURB65 score for the adverse medical outcome (p = 0.04) and tended to improve it for death (p = 0.06). The combination of ProET-1 and the PSI score did not significantly improve the prognostic value of the PSI score alone (AUC 0.71 [95%CI 0.62–0.76], p = 0.43 and AUC 0.75 [95%CI 0.68–0.81], p = 0.39).

When entering proET-1, procalcitonin, C-reactive protein, and each of the clinical severity scores in a multivariate logistic regression analysis, only proET-1 and a rise in one of the two risk scores were independent predictors of death and adverse medical outcome. Table 3 and Table 4 show the respective odds ratios and significance levels of all variables for both outcomes.

**Table 3:** Multivariate regression analysis for the prediction of death

**Prediction of mortality in multivariate regression analysis including the PSI score in patients with CAP (n = 281)**		
**Predictor**	**Odds ratio (95% CI)**	***P-value***

ProET-1	1.006 (1.0010–1.0011)	*0.018*
PSI	2.113 (1.308–1.412)	*0.002*
Procalcitonin	0.962 (0.901–1.026)	*0.246*
C-reactive protein	0.998 (0.993–1.001)	*0.274*

**Prediction of mortality in multivariate regression analysis including the CURB65 score in patients with CAP (n = 281)**		

**Predictor**	**Odds ratio (95% CI)**	***P-value***

ProET-1	1.007 (1.001–1.011)	0.010
CURB65	1.859 (1.214–1.847)	0.004
Procalcitonin	0.967 (0.910–1.026)	0.265
C-reactive protein	0.998 (0.993–1.001)	0.49

**Table 4:** Multivariate regression analysis for the prediction of adverse outcome

**Prediction of adverse medical outcome in multivariate regression analysis including the PSI score in patients with CAP (n = 281)**		
**Predictor**	**Odds ratio (95% CI)**	***P-value***

ProET-1	1.007 (1.002–1.001)	*0.004*
PSI	2.092 (1.434–3.051)	<*0.001*
Procalcitonin	0.976 (0.950–1.004)	*0.093*
C-reactive protein	1.001 (0.998–1.004)	*0.428*

**Prediction of adverse medical outcome in multivariate regression analysis including the CURB65 score in patients with CAP (n = 281)**		

**Predictor**	**Odds ratio (95% CI)**	***P-value***

ProET-1	1.007 (1.003–1.012)	0.001
CURB65	1.536 (1.096–2.153)	0.013
Procalcitonin	0.978 (0.952–1.003)	0.093
C-reactive protein	1.001 (0.998–1.004)	0.443

**Figure 6:**
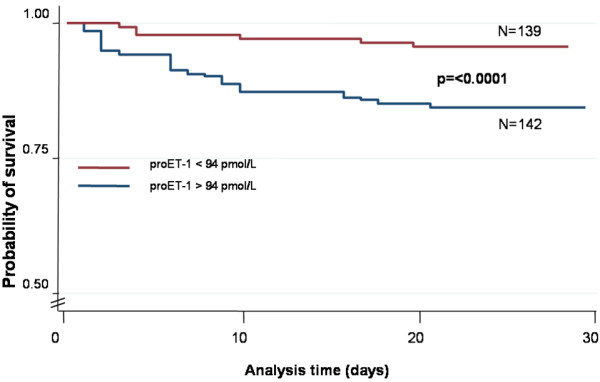
Kaplan Meier Survival curves showing the incidence of death in patients with proET-1 levels above and below 94 pmol/L. P = log rank test.

**Figure 7:**
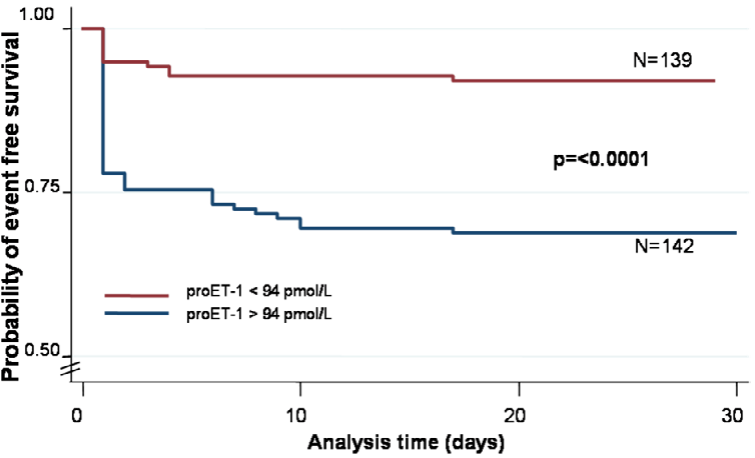
Kaplan Meier Survival curves showing the incidence of adverse medical outcome including death and/or ICU admission in patients with proET-1 levels above and below 94 pmol/L. P = log rank test.

Finally, to illustrate the capacity of proET-1 for risk assessment for patients admitted to the emergency room, we performed a comparison of survival (Figure 6) and adverse medical outcome (Figure 7) in patients with proET-1 below and above the optimal cut-off value of 94 pmol/l by Kaplan-Meier survival curves. Patients with proET-1 levels above the optimal cut-off had significantly lower survival rates and a higher risk for adverse medical outcome as compared to patients with levels below the cut-off of 94 pmol/L.

## Discussion

The main findings of this study are that circulating levels of ET-1 precursor peptides correlate with the severity of CAP, as assessed by the PSI and CURB65 scores, resolve during recovery of illness and predict the later finding of bacteraemia in patients with CAP. ProET-1 levels on admission are independent predictors of short term mortality and need for ICU admission with a moderate but superior prognostic accuracy as compared to commonly measured laboratory parameters. Importantly, proET-1 levels can improve the prognostic accuracy of the commonly used CURB65 score to predict adverse outcome.

ET-1 originates from a larger precursor peptide, which is first proteolytically processed to big ET-1 and further excised by the action of endothelin-converting enzyme [2]. In this study, we assessed the precursor fragment of ET-1, because proET-1 fragments are stable at room temperature and can be detected for hours after the cleavage in the circulation, in contrast to mature ET-1, which is eliminated within minutes and therefore escapes detection in clinical routine[10,11].

CAP accounts for almost 10% of mortality and morbidity in hospitalized patients in western countries [16]. The majority of patients in our study fulfilled the clinical criteria for sepsis. While different cytokines and toxins contribute to the extensive vasodilatation often seen in systemic infections, ET-1 as the most potent human vasoconstrictor counteracts these effects on the endothelial system assuring blood pressure homeostasis and blood supply to the individual organs [1,2]. However, accumulating evidence indicates that increased ET-1 levels as seen during sepsis rather contribute to the disturbance in blood pressure homeostasis causing multiorgan failure and eventually death [17]. Increased levels of mature ET-1 have been found in different experimental and clinical models of sepsis [5,12,18,19]. We found increased levels of proET-1 in our patients with CAP depending on disease severity and a decrease of proET-1 levels during recovery. In addition, we found a significant relationship of proET-1 with other markers of inflammation and infection. ProET-1 levels had a higher diagnostic accuracy with a high negative predictive value as compared to traditional biomarkers of infection (C-reactive protein and leukocyte count) and in the range of procalcitonin to exclude growth of bacteraemia in blood cultures.

Procalcitonin has been put forward as a useful marker for predicting disease severity and outcome in patients with pneumonia [20]. Notably, in our study procalcitonin levels correlated with CAP severity as assessed by the PSI and the CURB65 score, but procalcitonin did not reach the prognostic accuracy of proET-1 or the clinical scores. Based on our results, procalcitonin is rather a diagnostic tool able to guide decisions on antibiotic therapy, whereas proET-1 is a superior prognostic marker to predict severity of disease.

Sepsis is frequently associated with organ deterioration, especially renal and cardiopulmonary insufficiency [18]. Endothelin has been discussed as one of the main pathophysiological mechanisms underlying renal vasoconstriction during endotoxaemia [21]. Increased levels of ET-1 have been found during sepsis related renal insufficiency and infusion of ET-1 has been shown to reduce renal and splanchnic blood flow in healthy volunteers [2,22]. In addition, receptor antagonism of ET1 has been suggested to improve renal perfusion and function during sepsis [23]. Likewise, in our clinical study we found a significant correlation of ET-1 precursor peptides with creatinine and urea. ProET-1 levels may mirror both, the inflammatory cytokine response correlated with the severity of pneumonia, as well as the presence of disease-relevant comorbidities, namely renal dysfunction.

In the assessment and management of CAP, knowledge of prognostic factors is crucial to estimate the risk for adverse medical outcome and, thus the need for hospitalization. In this context, there is interest for new measurable biomarkers mirroring distinct pathogenetic mechanisms to predict severity and outcome in CAP. The utility of a biomarker in this context is defined by the degree it improves clinical decision making and adds timely information beyond that of readily available information from clinical examination [24]. Our retrospective study can not provide such information, but may help to provide a rationale for future prospective studies. The information of a biomarker may provide new insights into the pathophysiology and prognosis of the disease process facilitating risk stratification and monitoring of therapy as a surrogate outcome measure. In the future, a biomarker might help in delineating distinct populations of patients with discrete pathologies – a prerequisite to enable the targeted application of specific biologically rational therapies, i.e. ET-1 blocking agents like bosentan (Tracleer^®^) [6,8,24,8,26]. Our study demonstrates that ET-1 precursor peptides are of moderate diagnostic and prognostic utility as early and independent risk predictor for death and need for ICU admission in patients with CAP. In a multivariate logistic model the combination of proET-1 and the CURB65 risk scores significantly improved the prognostic accuracy of the new model. Because the prognostic utility of a single proET1 measurement was only moderate in this analysis, this biomarker should be imbedded in existing clinical risk scores and may improve their accuracy.

It is advisable to base the difficult task of prognostic assessment and treatment decisions on several and not only one parameter, each mirroring different pathophysiological aspects. The findings of this study support and extend other observational studies evaluating the benefit of novel biomarkers (e.g. natriuretic peptides, adrenomedullin and vasopressin precursor peptides) in the diagnostic assessment and risk stratification of patients with cardiovascular and infectious disease [27].

Some limitations should be considered in interpreting our results. First, this is a preplanned post-hoc analysis of the ProCAP study [13] and, thus, rather hypothesis-generating than definite. Second, the overall prognostic utility of proET1 with an AUC of 0.64 is rather low reflecting the considerable overlap between survivors and non-survivors, which narrows the clinical applicability of proET1. In addition, the number of events in this analysis is of only moderate size limiting the statistical power. For both reasons, our results need to be confirmed in future prospective studies with medical outcome as the primary endpoint.

## Conclusion

In conclusion, systemic ET-1 precursor peptides correlate with disease severity and short term outcome in patients with CAP. Intervention studies are needed to show whether proET-1 measurement improves risk prognostication and thus improves the clinical management of patients with CAP.

## Competing interests

BM and DS have served as consultants and received payments from Brahms (the manufacturer of procalcitonin assays) to attend meetings related to the trial and for travel expenses, speaking engagements, and research. PS and MCC received payments from Brahms for speaking engagements. NM and JS are employees of Brahms. All other co-authors declare that they have no competing interests.

## Authors' contributions

MCC and BM had the idea for the study and directed study design, data collection and analysis and writing of the report. PS analyzed the data and wrote the report. NM and JS did the analyses and helped in analyzing and writing of the report. DS, RB, CM and MT had substantial contributions in planning of the study, data collection, interpretation of data and/or writing of the manuscript. All authors have read and approved the final manuscript.

## Pre-publication history

The pre-publication history for this paper can be accessed here:


